# Theta activity discriminates high-level, species-specific body processes

**DOI:** 10.1162/imag_a_00150

**Published:** 2024-04-26

**Authors:** Jane Chesley, Lars Riecke, Juanzhi Lu, Rufin Vogels, Beatrice de Gelder

**Affiliations:** Department of Cognitive Neuroscience, Faculty of Psychology and Neuroscience, Maastricht University, Maastricht, The Netherlands; Laboratory for Neuro, and Psychophysiology, Department of Neurosciences, KU Leuven Medical School, Leuven, Belgium; Leuven Brain Institute, KU Leuven, Leuven, Belgium; Department of Computer Science, University College London, London, United Kingdom

**Keywords:** body processing, EEG, theta activity, oscillations

## Abstract

Among social stimuli that trigger rapid reactions, body images occupy a prominent place. Given that bodies carry information about other agents’ intentions, actions and emotional expressions, a foundational question concerns the neural basis of body processing. Previous functional magnetic resonance imaging (fMRI) studies have investigated this but were not yet able to clarify the time course and its functional significance. The present EEG study investigated the role of slow oscillatory cortical activity in body processing and species-specificity. Human participants viewed naturalistic images of human and monkey bodies, faces, and objects, along with mosaic-scrambled versions to control for low-level visual features. Analysis of event-related theta power (4–7 Hz) combined with data-driven methods revealed a strong, body-evoked neural response that is specific to human bodies and spans a widespread scalp region during a time window of 200–550 ms after the onset of the body image. Our results corroborate recent research proposing a species-specific cortical network of human body processing. We submit that this network may play an essential role in linking body processes to movement intentions.

## 1 Introduction

Social species vitally rely on information from their conspecifics to navigate the natural and social world. During social interactions, humans rapidly decode cues from the faces and bodies of others, which hold information relevant to identity, emotions, and actions. While the role of faces in regulating social interactions has been well-established (Freiwald et al., 2016;Powell et al., 2018;Schwiedrzik et al., 2015), evidence for a role of whole-body processing is still accumulating. Body-selective areas were first reported in the lateral occipitotemporal cortex, termed the extrastriate body area (EBA) and fusiform body area (Downing et al., 2001;Peelen & Downing, 2005). Further research has reported body-selective responses widespread throughout the brain in the posterior superior temporal sulcus (STS) (Candidi et al., 2015;Kret et al., 2011), temporoparietal junction, frontal cortex, and parietal motor areas (Pichon et al., 2009), as well as subcortical areas (de Gelder & Poyo Solanas, 2021;Poyo Solanas et al., 2020;Swann et al., 2012).

Furthermore, recent research combining advanced data-driven methods with 7-Tesla functional magnetic resonance imaging (fMRI) has revealed a large-scale network that is specifically selective for human body stimuli (Li et al., 2023). In that study, human participants viewed naturalistic videos of monkey and human faces, bodies, and objects, along with mosaic-scrambled versions to control for visual low-level features. Network analysis revealed two large-scale networks specifically selective for the processing of bodies in the lateral occipital cortex and right STS. Most notably, the right STS network was human-body-specific, as it showed high species selectivity for human versus monkey bodies. The aim of the present study is to further investigate the temporal properties of these species-specific body processes.

Previous lines of research using EEG have investigated the millisecond-precise timing of neural responses to bodies. With this method, event-related potential (ERP) studies have reported that, like faces, bodies are processed configurally, as shown by enhanced and delayed body-sensitive N170 ERPs to inverted versus normally oriented bodies (Stekelenburg & de Gelder, 2004). In addition, like faces, emotional information from body stimuli is rapidly encoded in early stages of visual processing, as differences between fearful and neutral body responses can emerge as early as 112 ms after stimulus onset (van Heijnsbergen et al., 2007). A body-specific ERP modulation has consistently been observed at 190 ms post-stimulus (N190) over occipito-temporal scalp regions in response to silhouettes of normal bodies compared to scrambled silhouettes (Thierry et al., 2006) as well as to headless naturalistic bodies compared to plants (Moreau et al., 2018;Taylor et al., 2010), providing further evidence for body-specific processes. Furthermore, intracranial local field potentials (iLFPs) have shown body-selective responses emerging from EBA at 190 ms post-stimulus, with a peak at 260 ms (Pourtois et al., 2007).

While EEG research has consistently shown body-related effects on stimulus-evoked broadband cortical responses, effects on oscillatory cortical responses have been investigated much less. Frequency-specific (narrow-band) oscillatory activity is thought to represent different areal and interareal processing mechanisms (Fries, 2009,^2015^;Wang, 2010), and modulations of oscillatory activity have been implicated in various cognitive functions like cognitive control, learning, memory, and action regulation (Cavanagh & Frank, 2014;Herweg et al., 2020;Trujillo & Allen, 2007). In particular, neural activity in the theta band (4–7 Hz) has been linked to body processes: differential theta activation has been observed over occipito-temporal and pre-frontal scalp regions for body versus face processing within 250–500 ms post-stimulus (Bossi et al., 2020). Moreover, these scalp regions have been shown to synchronize their theta activity in the aforementioned time window during the processing of visual body information during social interactions (Moreau et al., 2020). Furthermore, widespread theta activity has been observed throughout the brain within the first 400 ms of stimulus onset for self- and non-self body responses (Çelik et al., 2021). Overall, these findings suggest that oscillatory theta activity within 500 ms after body-image onset might play a relevant role in body processing.

An important methodological challenge in the study of neural representations of bodies is the control of low-level sensory information. Naturally, visual stimuli convey low- and high-level information. Low-level features include elementary visual information of luminance, contrast, and surface area, among others (Koch & Ullman, 1987;Veale et al., 2017). On the other hand, high-level features refer to semantic and categorical information, such as the identification of a stimulus as a “body,” “face,” or “object” (Groen et al., 2017;“High-level visual processing: Cognitive influences”, 2014). An effective approach to isolating the high-level processes in the brain is to include scrambled stimuli in the experimental design, as scrambled stimuli can preserve several low-level stimulus features while destroying higher-level information. Some ERP studies have used scrambled stimuli (van Heijnsbergen et al., 2007), but currently in the field, no oscillatory body research (see above) has adequately controlled for the contributions of low-level visual features with the use of scrambled body stimuli, leaving unclear whether their findings reflect visual or more abstract body representations. The present study aims to bridge this gap by including mosaic-scrambled stimuli that control for low-level features of luminance, contrast, and non-background area to better understand the role of oscillatory theta activity in high-level body processes.

In line with previous research on the role of slow oscillatory cortical activity in body processing, we hypothesized that theta activity plays a relevant role in the processing of static body stimuli. By using EEG and a data-driven approach, we first identified a strong theta response in a widespread, bi-lateral scalp region within 200–550 ms after the onset of visual categorical stimuli. Using an experimental design comprising category conditions (body, face, and object), visual controls (scrambled versions of the categorical stimuli), and species (human and monkey), we then tested whether these responses are human body-specific, while controlling for low-level visual features. Furthermore, based on the previous fMRI research suggesting a large-scale, species-specific network for human body processing (Li et al., 2023), we hypothesized that the high-level (scramble-controlled) representations of bodies would be species-specific, with a clear enhancement of human (versus monkey) body processing.

## 2 Methods

### 2.1 Ethics statement

Procedures were approved by the Ethical Committee of Maastricht University and were in accordance with the Declaration of Helsinki. Written, informed consent was obtained from participants prior to the experiment, and the study was conducted in accordance with local legislation and institutional requirements.

### 2.2 Participants

Thirty healthy, right-handed participants with normal or corrected-to-normal vision were recruited for this study. All participants reported no history of psychiatric or neurological disorders. Participants were compensated in either monetary vouchers (€7.5 per hour) or credit points (1 credit per hour). One participant’s data were excluded from the analysis because she/he presumably misunderstood the attention task (as shown by 0% accuracy); the remaining 29 participants had an average accuracy of 96 ± 4% (mean ± SD) (range = 85 – 100%). Hence, 29 participants’ data were included in the analysis (17 females; age range = 18-37 years; mean age = 23).

### 2.3 Stimuli

**Fig. 1.**
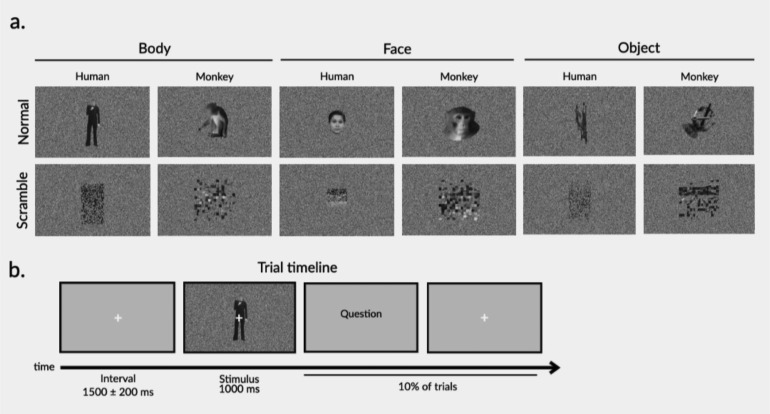
(a) Example stimuli for all conditions. The first row shows normal stimuli corresponding to the following conditions (left to right): human bodies, monkey bodies, human faces, monkey faces, artificial objects with the aspect ratio matched to human bodies, and artificial objects with the aspect ratio matched to monkey bodies. The second row shows the mosaic-scrambled versions of each respective normal stimulus. (b) Trial timeline.

Grayscale, naturalistic images of bodies, faces, and objects were used as stimuli in the experiment (Fig. 1a). Body and face stimuli were from a human or a monkey. Body stimuli had face information removed with Gaussian blurring. Object stimuli consisted of two sets of artificial objects (e.g., mechanical devices, vehicles, tools) and their aspect ratio matched either human bodies (set 1) or monkey bodies (set 2). Stimuli were embedded in a white noise background and presented centrally on the computer screen. The size of the stimuli was 9 * 9 degrees of visual angle for human faces, 9 * 20 degrees for human bodies and objects, and 16 * 16 degrees for monkey faces, bodies, and objects.

To control for the contribution of low-level visual features, mosaic-scrambled images were included. Mosaic-scrambled images destroyed the whole shape of each body/face/object stimulus but preserved the low-level features of luminance, contrast, and non-background area (Bognár et al., 2023). This resulted in a total of 12 experimental conditions (human/monkey * body/face/object * normal/scrambled). There were 10 different stimuli per condition, which resulted in 120 unique images.

All images were adapted from video stimuli used in previous studies on body and face processing (Bognár et al., 2023;Kret et al., 2011;Li et al., 2023;Zhu et al., 2013). The original videos were 1 second in duration, and the images for the present study were extracted from the midpoint (frame 30) of each original video (60 fps). The original body videos depicted either a human or a monkey performing naturalistic full-body movements, and the original face videos depicted either a human or a monkey performing naturalistic facial movements. The original human body and human face videos depicted both female and male actors dressed in black, performing expressions against a greenscreen background (Kret et al., 2011). The expressions included full-body or facial expressions of anger, fear, happiness, as well as neutral actions such as nose-pulling or coughing. The original monkey videos were recorded from rhesus monkeys from the Katholieke Universiteit Leuven monkey colony. The monkey body videos depicted full-body movements such as grasping, picking, turning, walking, threatening, throwing, wiping, and initiating jumping (Bognár et al., 2023). The monkey face videos depicted facial expressions such as chewing, lip-smacking, fear grin, and threat (Zhu et al., 2013). For all human and monkey videos, a variety of both emotional and neutral poses were included. The original object videos depicted non-rigid movements of computer-rendered artificial objects (created byhttps://garethwashere.tumblr.com) (Bognár et al., 2023).

Image extraction and stimulus presentation were programmed in MATLAB 2021a (The Mathworks, Natick, MA, USA) with the Psychophysics Toolbox extensions (Brainard, 1997;Kleiner et al., 2007;Pelli, 1997) as well as custom code.

### 2.4 Experimental design, task, and procedure

The experiment consisted of two experimental sessions, one of which presented images (see Stimuli) and the second of which presented videos of the same stimuli. The order of the two experimental sessions was randomized across participants. The present paper reports the methods, analysis, and results of the former, image-related experimental session; the latter was used for another project.

The main experiment employed a randomized design. There were four runs, all lasting around 6 minutes. During each run, 120 unique images (12 conditions × 10 stimuli; see Stimuli) were presented once in random order. This resulted in a total of four repetitions per stimulus and 40 repetitions per condition. Each trial began with a white fixation cross centered on a gray screen (Fig. 1b). To reduce the temporal expectancy of stimulus presentation, the intertrial interval was jittered at 1500 ms (1500 ± 200 ms). Participants viewed the images on a computer screen (1920 × 1080) at 65 cm from their eyes. A white fixation cross was centered and overlaid on each image. Participants were asked to focus their gaze on the fixation cross and focus their attention on each stimulus. To maintain attention, a question appeared on a random 10% of trials. The question asked about the content of the preceding stimulus (e.g., “What did the previous image show?”), and participants were asked to respond with a button press from a selection of “Body,” “Face,” “Object,” or “None of the above.”

### 2.5 EEG acquisition

EEG signals were acquired from 33 passive silver/silver chloride electrodes embedded in a fabric cap (EASYCAP GmbH) and arranged in accordance with the international 10–20 system. Scalp electrodes included: AFz, Fz, FCz, Cz, CPz, Pz, Oz, Fp1, Fp2, F3, F4, F7, F8, FC3, FC4, FT7, FT8, C3, C4, T7, T8, CP3, CP4, TP7, TP8, TP9, TP10, P3, P4, P7, P8, O1, and O2 (n = 33). EEG signals were amplified with a BrainVision amplifier (Brain Product GmbH, Germany) and recorded with BrainVision Recorder (Brain Product GmbH, Germany) at a sampling rate of 1000 Hz. Horizontal electrooculogram (HEOG) and vertical electrooculogram (VEOG) were recorded bipolarly from electrodes placed 1 cm from the eye. An online reference electrode was placed on the left mastoid, and an offline reference electrode was placed on the right mastoid. The ground electrode was placed on the forehead. Impedance was kept below 5 kΩ for all electrodes. EEG recordings took place in an electromagnetically shielded room.

### 2.6 EEG data preprocessing

EEG data were preprocessed and analyzed offline in MATLAB 2021a (The Mathworks, Natick, MA, USA) using the Fieldtrip Toolbox extensions (Oostenveld et al., 2011) as well as custom code. The signal was first segmented into trials from 500 ms pre-stimulus onset (image presentation) to 1500 ms post-stimulus. EEG data were re-referenced to the average of the signal at the left and right mastoids and downsampled to 250 Hz. Ocular movements were removed with Independent Component Analysis (ICA, logistic infomax ICA algorithm;Bell & Sejnowski, 1995); on average, 1.4 ± 0.5 (mean ± SD) eye movement-related components were visually identified and removed per participant. Single trials in which the peak amplitude exceeded 3 SD above/below the mean amplitude were rejected; on average, 91.2 ± 3.4% (mean ± SD) of trials were preserved per participant.

### 2.7 Time-frequency analyses

The preprocessed signal was filtered with a 1–30 Hz bandpass filter. Time-frequency power was computed for each trial by decomposing the signal with a complex Morlet wavelet transformation (frequency-bin size: 1 Hz, three cycles per time window; time-bin size: 50 ms). Baseline normalization was performed by log-transforming the power in the epoch of interest (0−1000 ms post-stimulus) relative to the average power in the pre-stimulus interval (−500 to−100 ms), separately for each frequency bin. The present analysis focuses on power in the theta (4–7 Hz) band, based on literature suggesting theta activity plays a role in body processing (see Introduction).

**Fig. 2.**
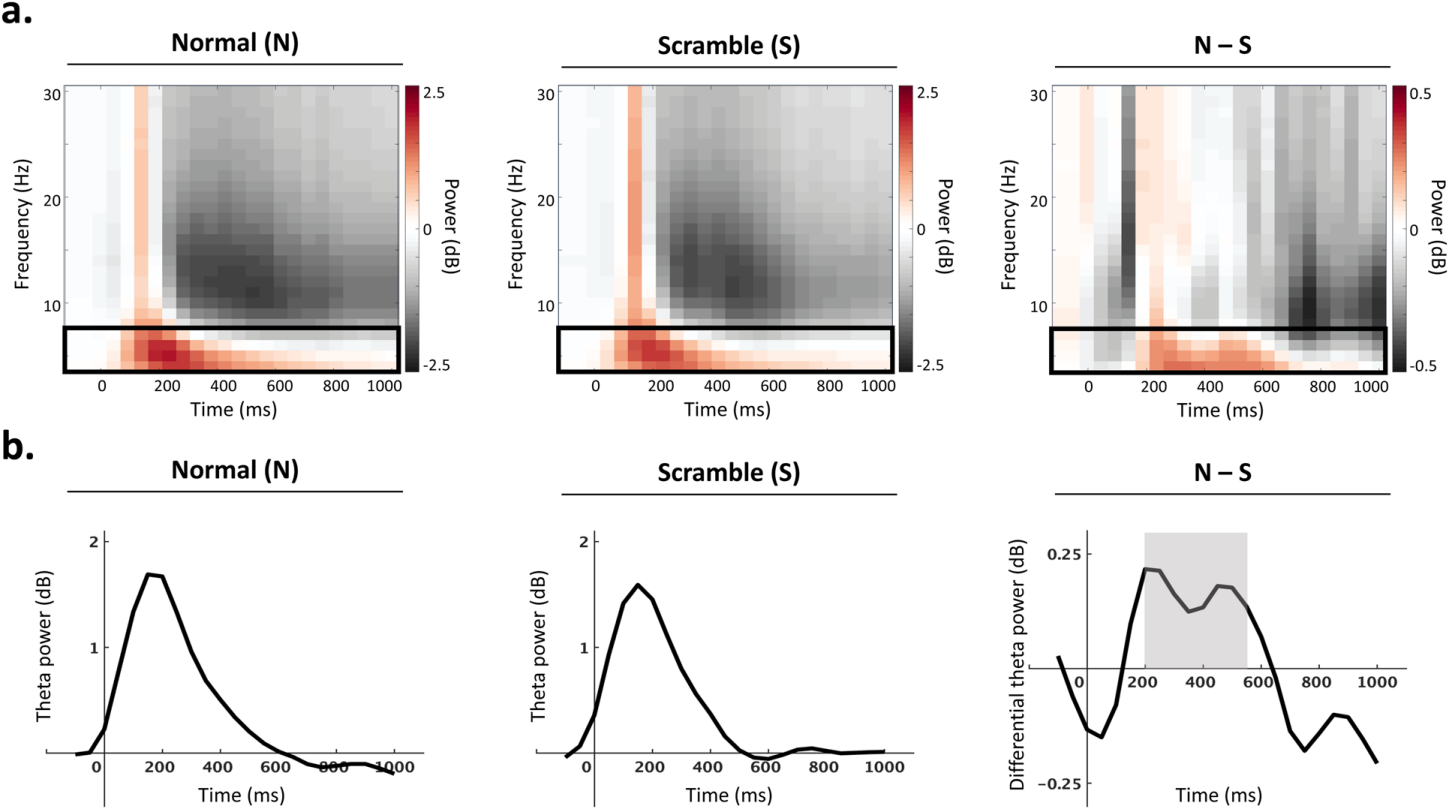
Time window selection. (a) Group-level power spectra computed across all electrodes for all normal (left) and all scramble (middle) conditions. Differential power (normal–scramble) is represented on the right panel. Theta activity (4–7 Hz) is indicated with a black box. Power relative to the pre-stimulus baseline is shown in decibels (dB) across time (ms) and frequency (Hz). (b) Time-series of theta power (dB) across conditions. The average theta power computed across all electrodes is shown for all normal (left) and all scramble (middle) conditions. Differential theta power (normal–scramble) is shown on the right panel, and the time window of interest (200–550 ms) is indicated with a grey box.

The time window of interest was selected based on previous literature suggesting body selectivity occurs in the theta band within 250–500 ms post-stimulus (Bossi et al., 2020), as well as inspection of the present data, which revealed two peaks between 200–550 ms post-stimulus for normal compared to scramble conditions (Fig. 2b; right panel). Based on this observation, the mean theta power during the time window (200–550 ms) was extracted at each electrode for all conditions.

### 2.8 Cluster-based permutation analyses

To extract scalp regions involved in visual object processing, non-parametric cluster-based permutation analysis was used to select groups of neighboring channels with a significant difference between normal and scramble conditions. With this data-driven method, the mean theta power during the time window of interest (200–550 ms) was pooled for all normal (human/monkey * body/face/object) and all scramble (human/monkey * body/face/object) conditions. For each electrode, normal and scramble conditions were compared by means of a t-test (one-sided; normal > scramble). Neighboring electrodes (minimum group size = 2) with t-values exceeding a threshold of p < 0.05 were defined as clusters. Cluster-level test statistics were calculated by summing the t-values within each cluster. To test the statistical significance of the clusters, Monte Carlo permutation tests were run (N = 2000 permutations) to obtain a null distribution of cluster-level test statistics. Cluster-level test statistics computed from observed data were statistically compared to the reference distribution. Clusters with a probability below a critical alpha level of 0.05 were deemed significant.

**Fig. 3.**
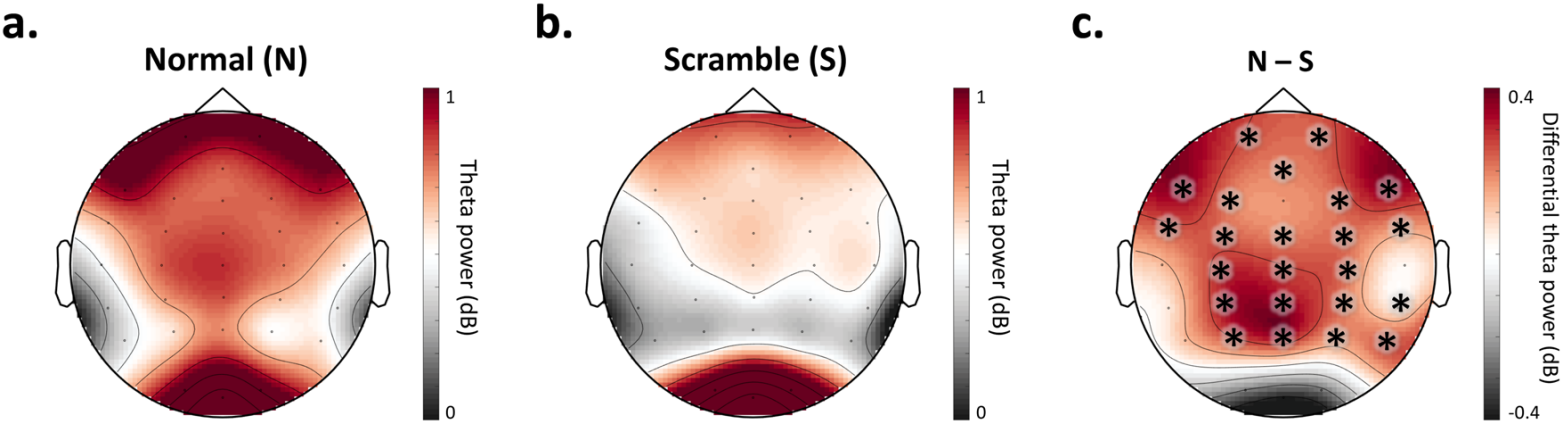
Channel selection. Theta power (4–7 Hz) during the time window of interest (200–550 ms post-stimulus) for all normal (a) and all scramble (b) conditions. The difference in power (normal–scramble) is represented in (c). Power is shown in decibels (dB). Cluster-based permutation analysis revealed significant differences (p = 0.001) between all normal (a) and all scramble (b) conditions within a cluster of 23 electrodes: AFz, FCz, Cz, CPz, Pz, Fp1, Fp2, F3, F4, F7, F8, FC3, FC4, FT7, FT8, C3, C4, CP3, CP4, TP10, P3, P4, and P8, indicated with asterisks in (c).

Cluster-based permutation analysis of theta power during the time window of interest (200–550 ms) revealed a significant difference between normal and scramble conditions in a widespread, bi-lateral cluster, which included 23 electrodes: AFz, FCz, Cz, CPz, Pz, Fp1, Fp2, F3, F4, F7, F8, FC3, FC4, FT7, FT8, C3, C4, CP3, CP4, TP10, P3, P4, and P8 (p = 0.001) (Fig. 3). From this point forward, this group of electrodes is referred to as the scalp region of interest (ROI) and is utilized for further analyses.

### 2.9 Theta power difference

To control for the neural processing of low-level visual features, the difference between normal and scramble conditions was computed for each category. Specifically, the subject-level mean theta activity (200–550 ms; ROI) for each scramble condition was subtracted from the respective activity for each normal condition: human body (normal–scramble); monkey body (normal–scramble); human face (normal–scramble); monkey face (normal–scramble); human object (normal–scramble); and monkey object (normal–scramble). The resulting differential activity was deemed to represent theta activity related to high-level neural processes and was further analyzed.

### 2.10 Statistical analyses

Statistical analyses were performed using IBM SPSS Statistics 28 (IBM Corp., Armonk, NY, USA). A repeated-measures 2×3 ANOVA (Species: human/monkey * Category: body/face/object) was applied to the mean theta power difference (normal–scramble). Statistical differences below p < 0.05 were considered significant. To control for type I errors, a False Discovery Rate (FDR) correction was applied to correct for multiple comparisons; only corrected p-values are reported.

## 3 Results

**Fig. 4.**
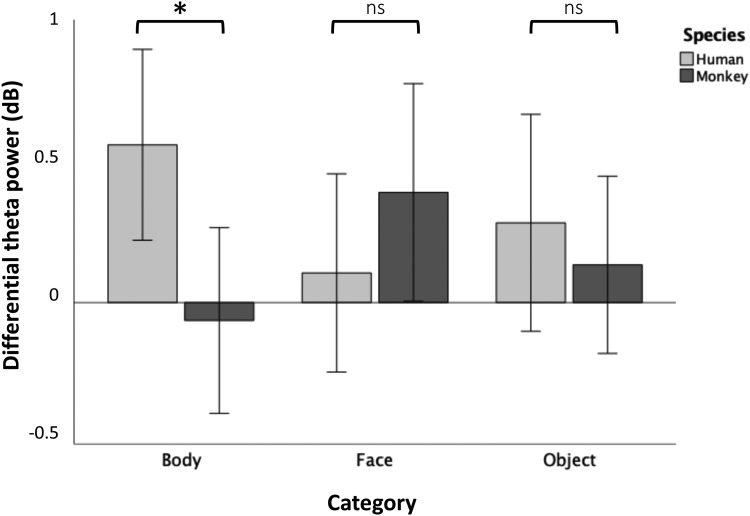
Means of differential theta power (normal–scramble) during the time window of interest (200–550 ms post-stimulus), calculated over the ROI for each condition. *p < 0.05. n.s., non-significant.

**Fig. 5.**
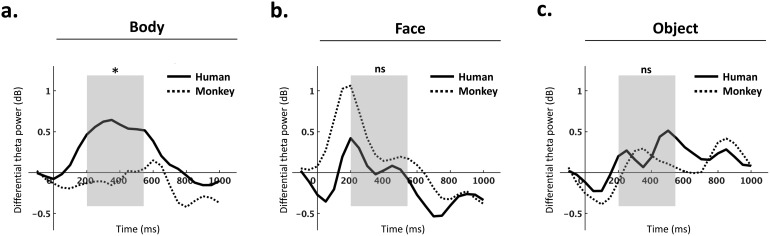
Time-series of differential theta power (normal–scramble) calculated over the ROI, shown separately for body stimuli (a), face stimuli (b), and object stimuli (c). Solid lines represent human stimuli, and dashed lines represent monkey stimuli. The time window of interest (200–550 ms) is indicated with a grey box. Differential theta power is shown in decibels (dB), and time is shown in milliseconds (ms). Repeated-measures ANOVA revealed a significant difference between human body (N-S) and monkey body (N-S) conditions in the time window of interest (p < 0.05) (a), as indicated with an asterisk. This species effect was not significant (ns) among face (b) or object (c) stimuli.

The interaction effect of species*category on differential theta power (normal–scramble) was significant (*F*(2,28) = 4.72,*p*= 0.038,*η_p_^2^*= 0.14). The main effect of species (*F*(1,28) = 1.29,*p*= 0.4,*η_p_^2^*= 0.04) and the main effect of category (*F*(2,28) = 0.03,*p*= 0.971,*η_p_^2^*< 0.001) were not significant. To investigate this interaction effect, three paired-samples t-tests were performed to compare the effect of species on differential theta power (normal–scramble) corresponding to body stimuli, face stimuli and object stimuli, respectively. There was a statistically significant difference in differential theta power between human bodies (M = 0.56, SD = 0.89) and monkey bodies (M = -0.06, SD = 0.86;*t*(28) = 2.78,*p*= 0.014) (Figs. 4-5). Importantly, this species effect was limited to body processing, as no corresponding difference in differential theta power could be found between human faces (M = 0.1, SD = 0.92) and monkey faces (M = -0.39, SD = 1.01;*t*(28) = -1.32,*p*= 0.148), nor between human objects (M = 0.28, SD = 1.01) and monkey objects (M = -0.13, SD = 0.82;*t*(28) = -0.66,*p*= 0.259).

### 3.1 Post-hoc, exploratory analyses and results

**Fig. 6.**
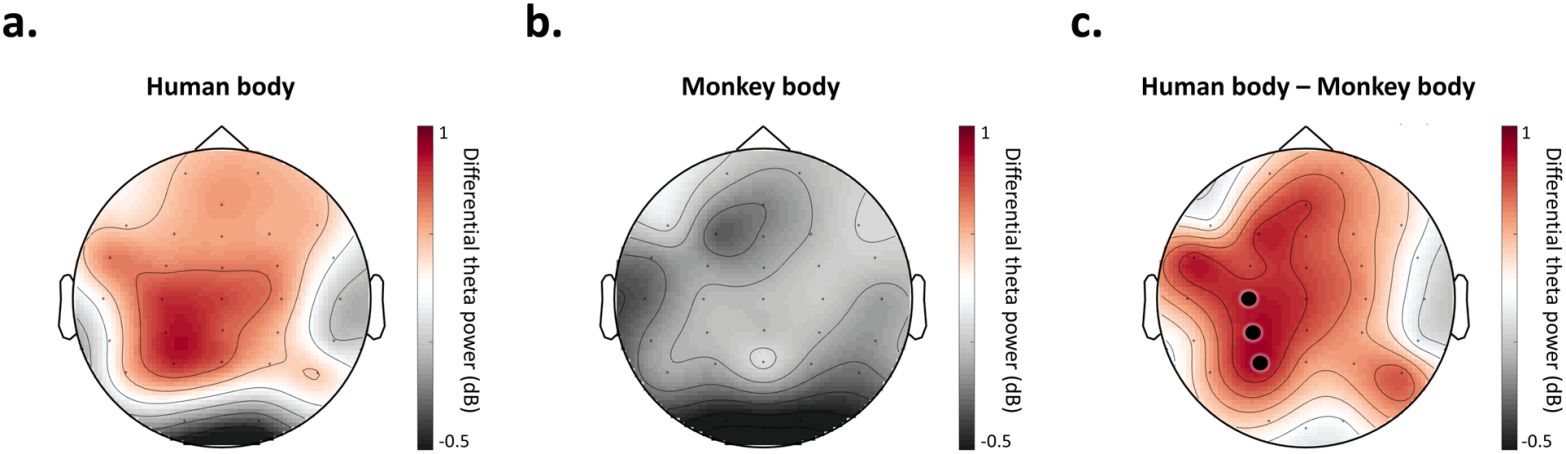
Group-level topography of differential theta power (normal – scramble) during the time window of interest for human body stimuli (a) and monkey body stimuli (b), as well as their difference (c). The strongest difference was observed at positions C3, CP3, and P3, indicated with black points in (c).

Post-hoc, exploratory analyses were run to further characterize the observed effect of species on body processing. First, to explore the spatial distribution of the effect, the effect size (Cohen’s d) of differential theta power between human body and monkey body stimuli during the time-window of interest was computed for each individual channel (N = 33). Three channels were observed at the 10^th^percentile: C3, CP3, and P3, indicating the maximum difference between human and monkey body conditions was observed within a left-sided sub-cluster of the original ROI (Fig. 6).

**Fig. 7.**
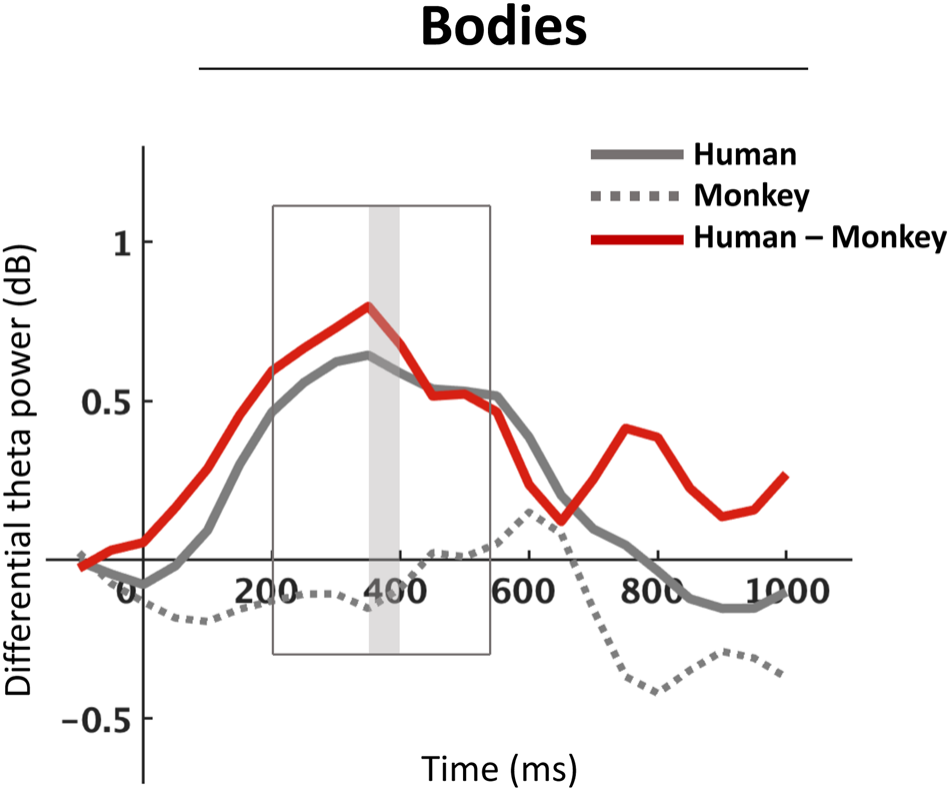
Time-series of differential theta power (normal–scramble) calculated over the ROI, shown separately for human body stimuli (solid gray line), monkey body stimuli (dashed gray line), and their difference (red line). The waveforms corresponding to human body and monkey body stimuli are the same as inFigure 5a. The original time window of interest is outlined (200–550 ms). The gray box shows the time-window showing the strongest difference between scramble-controlled human and monkey body conditions (350–400 ms).

Second, to further characterize the temporal profile of the effect, subject-level mean differential theta power in the ROI was computed for human body and monkey body conditions, separately for each time point during the interval 0 to 1000 ms post-stimulus in 50 ms increments (N = 21 time points). Visual inspection of the differential theta-power waveforms revealed that the species effect started building up rapidly after the onset of the visual stimulation and reached its maximum during the time window of interest at around ~350 ms after stimulation onset. After this window, the effect briefly emerged again around ~750 ms, but less strongly than during the earlier main window. To further explore the temporal profile of the effect, the effect size (Cohen’s d) of differential theta power between human body and monkey body stimuli within the ROI was computed for each individual time point (N = 21). Two time points were observed at the 10^th^percentile: 350 and 400 ms, indicating the maximum difference between human and monkey body conditions was observed within this window (Fig. 7).

ERP analyses were performed to further investigate whether the identified oscillatory effect might reflect evoked or induced activity. The same analysis pipeline was applied as for the time-frequency analysis (seeSupplementary Analyses). We found no significant difference in ERP amplitude between human bodies and monkey bodies (see Supplementary Materials;Fig. S1), mismatching the results based on differential theta power. This indicates that the species effect on body processing was reflected in theta oscillations rather than phase-locked activity.

Finally, to investigate whether the effect was specific to the theta-band, we applied the analysis pipeline to alpha- (8–12 Hz) and beta-band (13–30 Hz) power (seeSupplementary Analyses). There was no significant difference between normal and scramble conditions at any clusters of electrodes during the time window of interest in the alpha- or beta-bands (see Supplementary Materials;Fig. S2); no region of interest representing visual object-level processing could be identified.

## 4 Discussion

Our goal was to investigate the temporal and spectral patterns of species-specific body processes. Given recent fMRI findings proposing a large-scale, human-body-specific network (Li et al., 2023), we hypothesized that human body processing is accompanied by a temporary enhancement of theta activity compared to monkey body processing. In line with this hypothesis, we found a clear effect of species on visual object-level processing that was specific to bodies. More specifically, we found a significant enhancement of the neural representations of human (versus monkey) bodies, and most notably, this species effect was not present among face or object stimuli (Figs. 4-5). This body-specific process affected low-frequency (theta; 4–7 Hz) activity possibly originating from widespread brain regions (Fig. 3c) during a time window of 200–550 ms post-stimulus (Fig. 5a). Finally, we found this process may reflect induced activity in the theta band, and it did not extend to alpha (8–12 Hz) or beta (13–30 Hz) frequencies (see Supplementary Materials;Figs. S1–S2). Our findings corroborate previous findings linking oscillatory theta activity to body processing (Bossi et al., 2020;Çelik et al., 2021;Moreau et al., 2020). More importantly, our findings show a specificity of body processing for species, which is consistent with recent fMRI research suggesting body processing is species-specific and topographically widespread beyond EBA (Çelik et al., 2021;Li et al., 2023).

Numerous EEG studies on body processing have focused on the analysis of ERPs, and there is substantial evidence for a body-evoked cortical response at 190 ms (N190) post-stimulus (Moreau et al., 2018;Peelen & Downing, 2007;Taylor et al., 2010;Thierry et al., 2006). On the other hand, oscillatory cortical responses in the context of body processing have been investigated much less, yet the method is powerful in aiding our understanding of cognitive processes reflecting endogenous, non-phase-locked activity, which is attenuated in ERP analyses (Cohen, 2014;Luck, 2014). Furthermore, modulations of frequency-specific activity have been consistently implicated in cognitive functions (Cavanagh & Frank, 2014;Herweg et al., 2020;Trujillo & Allen, 2007), but only recently have oscillations been investigated in the context of body processing. Recent research has compared theta activation for body versus face processing (Bossi et al., 2020) and self- versus non-self-bodies (Çelik et al., 2021), as well as for body processing amid social interactions (Moreau et al., 2020). Yet, none of these oscillatory studies have investigated species-specific effects, which marks the novelty and aim of the present study.

Our channel-wise exploration of species-specific body processing revealed maximal differences in a left-sided cluster (channels C3, CP3, and P3;Fig. 6). This finding is in line with previous research showing a left-sided effect in the theta band for upright versus inverted bodies (Bossi et al., 2020); this potential left-sided bias is unclear and requires future investigation. Furthermore, an important methodological limitation of the present study is the low spatial specificity of EEG (Luck, 2014). To infer which specific brain regions are the source of the electrical activity recorded with scalp EEG, additional methods for source localization must be applied (Michel & He, 2019). Future research should implement such techniques to understand the precise cortical sources of the oscillatory effects observed in the present study.

In addition, our time point-wise exploration of the precise timing of the species-specific theta effect suggested that the effect built up rapidly after the onset of visual stimulation and reached its maximum around 350–400 ms (Fig. 7). As our measure of theta activity blended ongoing and phase-locked oscillatory activity, we attempted to separate these two; to this end we analyzed ERPs, a measure of purely phase-locked activity. However, unlike the theta activity-based results, the species-specific effect for bodies in the defined scalp region and time window was not significant in the ERP (see Supplementary Materials;Fig. S1), which may suggest the effect operates on higher-order, top-down processes that are not strictly phase-locked to the visual stimulus (David et al., 2006;Herrmann et al., 2014). However, it is important to note that there is an ongoing debate about whether oscillatory activity primarily reflects top-down processes (David et al., 2006;Herrmann et al., 2014) or bottom-up processes (Busch et al., 2006;Jia et al., 2022). In line with the former view, our results may suggest the species-specific effect reflects higher-order processes; however, future research on these theoretical frameworks is warranted to confidently disentangle the two processes.

Finally, we investigated whether species-specific body processing was reflected in other oscillatory frequency bands, and we did not find any corresponding effect in these oscillatory bands (see Supplementary Materials;Fig. S2). This further corroborates previous research suggesting oscillatory theta activity plays a relevant role in body processing (Bossi et al., 2020;Çelik et al., 2021;Moreau et al., 2020). Nevertheless, it is possible that oscillatory activity in other frequency bands may also play a role in body processing, and an interesting future direction can investigate those effects in other time-windows.

So far, species-specificity is not fully understood in the nonhuman primate brain. There is consistent evidence for body-selective patches in the macaque temporal cortex (for a review, seeVogels, 2022). In addition, single-unit recordings directly from body-selective patches in the macaque STS revealed differences between bodies and non-bodies, as well as between humans and monkeys, indicating effects at multiple processing levels (Kumar & Vogels, 2019). A follow-up to the present study can address the generalizability of our findings to nonhuman primate observers of primate bodies. On the same note, in the present study, human participants were likely more familiar with human images than monkey images, implying that the observed species effect might be confounded by familiarity. This possible alternative interpretation could be tested in a follow-up study, including nonhuman primate observers of nonhuman primate bodies. However, an explanation of our result on body images solely based on familiarity is implausible, given that we found no corresponding effect for face images. Thus, we would expect to find that in the nonhuman primate cortex, theta activity is enhanced in response to images of monkey versus human bodies. Another future direction can integrate the findings of human and monkey studies to create a comprehensive model of body processing in the brain. Recently, neural network models (Kumar et al., 2023) and theoretical frameworks (de Gelder & Poyo Solanas, 2021) for body processing have been proposed, but we do not have a complete understanding of the neural representations of bodies (Vogels, 2022).

The aim of the present study focused on the neural basis of body processing. While the underpinnings of face processing have been studied for several decades (Freiwald et al., 2016;Powell et al., 2018;Schwiedrzik et al., 2015), research on whole-body processing is still accumulating (de Gelder & Poyo Solanas, 2021;Taubert et al., 2022). However, there is no uncertainty that faces and bodies are similarly important in daily life and often convey crucial information for social communication (de Gelder, 2009). A large body of research has shown temporal and spatial differences in the neural representations of faces and bodies (Downing et al., 2001;Peelen & Downing, 2007;Poyo Solanas et al., 2018;Stekelenburg & de Gelder, 2004;Thierry et al., 2006). The present study included face stimuli to establish body specificity of the species effect, thus ruling out a generic, category-unrelated effect. The results demonstrate clear body-specific processes reflected in oscillatory activity and, furthermore, corroborate the findings of species-specificity in body processing (Li et al., 2023). In our investigation of body processing, we also found a species effect that affected specifically face processing reflected in ERPs (see Supplementary Materials;Fig. S1). Without doubt, future research is needed to better understand the dynamics of the integration of face and body information.

A central question concerns the functional significance of theta oscillations associated with species-specific body processing. Recent reports of theta oscillations offer some interesting and suggestive indications. Studies involving simple conflict paradigms have long suggested theta activity is a mechanism for cognitive control (for a review, seeCavanagh & Frank, 2014). More recently, theta activity was measured in response to approach-avoidance conflicts for the first time, and findings showed a direct relationship between midfrontal theta activation and approach-avoidance conflicts (Lange et al., 2022). A different but potentially highly relevant role of theta oscillations concerns perception-movement initiation at early stages. For example, oscillations in the theta-band may play an important role in combining in a common temporal reference frame visual perception and motor intention (Tomassini et al., 2017). Furthermore, studies on body perception have systematically shown that observing whole body actions is associated with activity in premotor and motor areas (de Gelder et al., 2010;Goldberg et al., 2014;Grèzes et al., 2007;Pichon et al., 2009). The theta effects observed in the present study may be linked to visual body perception in combination with processes related to movement intention. This pattern may have been driven by the inclusion of threatening stimuli, reflecting well-established processes seen in the theta band and related to cognitive control (for a review, seeCavanagh & Frank, 2014). The images used in the present design were selected to have a wide range of body expressions, including neutral expressions as well as emotional expressions depicting defensive actions (fear) and aggressive actions (anger), among others. This does not reduce the importance of the species-specific effect, as the monkey stimulus set equally included neutral and emotionally expressive actions but did not show a similar theta response. Taken together, the observed theta band activity provides clear suggestions for the underlying functional significance of species-specificity.

Another key feature of bodies is dynamics. In daily life, people who interact are not stationary but rather they are, to some degree, always moving. Emerging research using dynamic body stimuli has shown body- and motion-selective processes may be integrated (Kumar et al., 2023;Raman et al., 2023). While the present study used static images, future research should implement dynamic videos to understand the full extent of oscillatory representations of social interactions beyond static object recognition.

## Supplementary Material

Supplementary Material

## Data Availability

The data and code that support the findings of this study are available on request from the corresponding author (Beatrice de Gelder), pending approval from the researcher’s local ethics committee and a formal data-sharing agreement.

## References

[b1] ( 2014 ). High-level visual processing: Cognitive influences . In Kandel E. R. , Schwartz J. H. , Jessell T. M. , Siegelbaum S. A. , Hudspeth A. J. , & Mack S. (Eds.), Principles of neural science ( 5th ed.). McGraw Hill . https://neurology.mhmedical.com/content.aspx?bookid=1049&sectionid=59138656

[b2] Bell , A. J. , & Sejnowski , T. J. ( 1995 ). An information-maximization approach to blind separation and blind deconvolution . Neural Computation , 7 ( 6 ), 1129 – 1159 . 10.1162/neco.1995.7.6.1129 7584893

[b3] Bognár , A. , Raman , R. , Taubert , N. , Zafirova , Y. , Li , B. , Giese , M. , de Gelder , B. , & Vogels , R. ( 2023 ). The contribution of dynamics to macaque body and face patch responses . NeuroImage , 269 , 119907 . 10.1016/j.neuroimage.2023.119907 36717042 PMC9986793

[b4] Bossi , F. , Premoli , I. , Pizzamiglio , S. , Balaban , S. , Ricciardelli , P. , & Rivolta , D. ( 2020 ). Theta- and gamma-band activity discriminates face, body and object perception . Frontiers in Human Neuroscience , 14 , 74 . 10.3389/fnhum.2020.00074 32226369 PMC7080986

[b5] Brainard , D. H. ( 1997 ). The psychophysics toolbox . Spatial Vision , 10 ( 4 ), 433 – 436 . 10.1163/156856897X00357 9176952

[b6] Busch , N. A. , Schadow , J. , Fründ , I. , & Herrmann , C. S. ( 2006 ). Time-frequency analysis of target detection reveals an early interface between bottom-up and top-down processes in the gamma-band . NeuroImage , 29 ( 4 ), 1106 – 1116 . 10.1016/j.neuroimage.2005.09.009 16246588

[b7] Candidi , M. , Stienen , B. M. , Aglioti , S. M. , & de Gelder , B. ( 2015 ). Virtual lesion of right posterior superior temporal sulcus modulates conscious visual perception of fearful expressions in faces and bodies . Cortex , 65 , 184 – 194 . 10.1016/j.cortex.2015.01.012 25835522

[b8] Cavanagh , J. F. , & Frank , M. J. ( 2014 ). Frontal theta as a mechanism for cognitive control . Trends in Cognitive Sciences , 18 ( 8 ), 414 – 421 . 10.1016/j.tics.2014.04.012 24835663 PMC4112145

[b9] Çelik , S. , Doğan , R. B. , Parlatan , C. S. , & Güntekin , B. ( 2021 ). Distinct brain oscillatory responses for the perception and identification of one’s own body from other’s body . Cognitive Neurodynamics , 15 ( 6 ), 609 – 620 . 10.1007/s11571-020-09660-z 34367363 PMC8286911

[b10] Cohen , M. X. ( 2014 ). Analyzing neural time series data: Theory and practice . The MIT Press . 10.7551/mitpress/9609.001.0001

[b11] David , O. , Kilner , J. , & Friston , K. ( 2006 ). Mechanisms of evoked and induced responses in MEG/EEG . NeuroImage , 31 , 1580 – 1591 . 10.1016/j.neuroimage.2006.02.034 16632378

[b12] de Gelder , B. ( 2009 ). Why bodies? Twelve reasons for including bodily expressions in affective neuroscience . Philosophical Transactions of the Royal Society B , 364 ( 1535 ), 3475 – 3484 . 10.1098/rstb.2009.0190 PMC2781896 19884142

[b13] de Gelder , B. , & Poyo Solanas , M. ( 2021 ). A computational neuroethology perspective on body and expression perception . Trends in Cognitive Sciences , 25 ( 9 ), 744 – 756 . 10.1016/j.tics.2021.05.010 34147363

[b14] de Gelder , B. , Van den Stock , J. , Meeren , H. K. M. , Sinke , C. B. A. , Kret , M. E. , & Tamietto , M. ( 2010 ). Standing up for the body: Recent progress in uncovering the networks involved in the perception of bodies and bodily expressions . Neuroscience & Biobehavioral Reviews , 34 ( 4 ), 513 – 527 . 10.1016/j.neubiorev.2009.10.008 19857515

[b15] Downing , P. E. , Jiang , Y. , Shuman , M. , & Kanwisher , N. ( 2001 ). A cortical area selective for visual processing of the human body . Science , 293 ( 5539 ), 2470 – 2473 . 10.1126/science.1063414 11577239

[b16] Freiwald , W. , Duchaine , B. , & Yovel , G. ( 2016 ). Face processing systems: From neurons to real-world social perception . Annual Review of Neuroscience , 39 ( 1 ), 325 – 346 . 10.1146/annurev-neuro-070815-013934 PMC5345271 27442071

[b17] Fries , P. ( 2009 ). Neuronal gamma-band synchronization as a fundamental process in cortical computation . Annual Review of Neuroscience , 32 , 209 – 224 . 10.1146/annurev.neuro.051508.135603 19400723

[b18] Fries , P. ( 2015 ). Rhythms for cognition: Communication through coherence . Neuron , 88 ( 1 ), 220 – 235 . 10.1016/j.neuron.2015.09.034 26447583 PMC4605134

[b19] Goldberg , H. , Preminger , S. , & Malach , R. ( 2014 ). The emotion-action link? Naturalistic emotional stimuli preferentially activate the human dorsal visual stream . NeuroImage , 84 , 254 – 264 . 10.1016/j.neuroimage.2013.08.032 23994457

[b20] Grèzes , J. , Pichon , S. , & de Gelder , B. ( 2007 ). Perceiving fear in dynamic body expressions . NeuroImage , 35 ( 2 ), 959 – 967 . 10.1016/j.neuroimage.2006.11.030 17270466

[b21] Groen , I. I. A. , Silson , E. H. , & Baker , C. I. ( 2017 ). Contributions of low- and high-level properties to neural processing of visual scenes in the human brain . Philosophical transactions of the Royal Society of London. Series B, Biological sciences , 372 ( 1714 ), 20160102 . 10.1098/rstb.2016.0102 28044013 PMC5206270

[b22] Herrmann , C. S. , Rach , S. , Vosskuhl , J. , & Strüber , D. ( 2014 ). Time–frequency analysis of event-related potentials: A brief tutorial . Brain Topography , 27 , 438 – 450 . 10.1007/s10548-013-0327-5 24194116

[b23] Herweg , N. A. , Solomon , E. A. , & Kahana , M. J. ( 2020 ). Theta oscillations in human memory . Trends in Cognitive Sciences , 24 ( 3 ), 208 – 227 . 10.1016/j.tics.2019.12.006 32029359 PMC8310425

[b24] Jia , J. , Fan , Y. , & Luo , H. ( 2022 ). Alpha-band phase modulates bottom-up feature processing . Cerebral Cortex , 32 ( 6 ), 1260 – 1268 . 10.1093/cercor/bhab291 34411242

[b25] Kleiner , M. , Brainard , D. , & Pelli , D. ( 2007 ). What’s new in Psychtoolbox-3 ? Perception , 36 ( 14 , ECVP Abstract Supplement), 1 – 16 . https://hdl.handle.net/11858/00-001M-0000-0013-CC89-F

[b26] Kret , M. E. , Pichon , S. , Grèzes , J. , & de Gelder , B. ( 2011 ). Similarities and differences in perceiving threat from dynamic faces and bodies: An fMRI study . NeuroImage , 54 ( 2 ), 1755 – 1762 . 10.1016/j.neuroimage.2010.08.012 20723605

[b27] Koch , C. , Ullman , S. ( 1987 ). Shifts in selective visual attention: Towards the underlying neural circuitry . In Vaina L. M. (Ed.), Matters of intelligence. Synthese library (Vol. 188 ). Springer . 10.1007/978-94-009-3833-5_5 3836989

[b28] Kumar , P. , Taubert , N. , Raman , R. , Bognár , A. , Nejad , G. G. , Vogels , R. , & Giese , M. A. ( 2023 ). Neurodynamical model of the visual recognition of dynamic bodily actions from silhouettes . In Iliadis L. , Papaleonidas A. , Angelov P. , & Jayne C. (Eds.), Artificial neural networks and machine learning—ICANN 2023. ICANN 2023. Lecture notes in computer science (Vol. 14255 ). Springer . 10.1007/978-3-031-44210-0_43

[b29] Kumar , S. , & Vogels , R. ( 2019 ). Body patches in inferior temporal cortex encode categories with different temporal dynamics . Journal of Cognitive Neuroscience , 31 ( 11 ), 1699 – 1709 . 10.1162/jocn_a_01444 31274393

[b30] Lange , L. , Rommerskirchen , L. , & Osinsky , R. ( 2022 ). Midfrontal theta activity is sensitive to approach–avoidance conflict . Journal of Neuroscience , 42 ( 41 ), 7799 – 7808 . 10.1523/JNEUROSCI.2499-21.2022 36414005 PMC9581558

[b31] Li , B. , Poyo Solanas , M. , Marrazzo , G. , Raman , R. , Taubert , N. , Giese , M. , Vogels , R. , & de Gelder , B. ( 2023 ). A large-scale brain network of species-specific dynamic human body perception . Progress in Neurobiology , 221 , 102398 . 10.1016/j.pneurobio.2022.102398 36565985

[b32] Luck , S. J. ( 2014 ). An introduction to the event-related potential technique ( 2nd ed.). The MIT Press . https://mitpress.mit.edu/9780262525855/an-introduction-to-the-event-related-potential-technique/

[b33] Michel , C. M. , & He , B. ( 2019 ). EEG source localization . In Levin K. H. & Chauvel P. (Eds.), Handbook of clinical neurology (Vol. 160 , pp. 85 – 101 ). Elsevier . 10.1016/B978-0-444-64032-1.00006-0 31277878

[b34] Moreau , Q. , Candidi , M. , Era , V. , Tieri , G. , & Aglioti , S. M. ( 2020 ). Midline frontal and occipito-temporal activity during error monitoring in dyadic motor interactions . Cortex , 127 , 131 – 149 . 10.1016/j.cortex.2020.01.020 32197149

[b35] Moreau , Q. , Pavone , E. F. , Aglioti , S. M. , & Candidi , M. ( 2018 ). Theta synchronization over occipito-temporal cortices during visual perception of body parts . European Journal of Neuroscience , 48 ( 9 ), 2826 – 2835 . 10.1111/ejn.13782 29178557

[b36] Oostenveld , R. , Fries , P. , Maris , E. , & Schoffelen , J. M. ( 2011 ). FieldTrip: Open source software for advanced analysis of MEG, EEG, and invasive electrophysiological data . Computational Intelligence and Neuroscience , 2011 , 156869 . 10.1155/2011/156869 21253357 PMC3021840

[b37] Peelen , M. V. , & Downing , P. E. ( 2005 ). Selectivity for the human body in the fusiform gyrus . Journal of Neurophysiology , 93 ( 1 ), 603 – 608 . 10.1152/jn.00513.2004 15295012

[b38] Peelen , M. V. , & Downing , P. E. ( 2007 ). The neural basis of visual body perception . Nature Reviews Neuroscience , 8 ( 8 ), 636 – 648 . 10.1038/nrn2195 17643089

[b39] Pelli , D. G. ( 1997 ). The VideoToolbox software for visual psychophysics: Transforming numbers into movies . Spatial Vision , 10 ( 4 ), 437 – 442 . 10.1163/156856897X00366 9176953

[b40] Pichon , S. , de Gelder , B. , & Grèzes , J. ( 2009 ). Two different faces of threat: Comparing the neural systems for recognizing fear and anger in dynamic body expressions . NeuroImage , 47 ( 4 ), 1873 – 1883 . 10.1016/j.neuroimage.2009.03.084 19371787

[b41] Pourtois , G. , Peelen , M. V. , Spinelli , L. , Seeck , M. , & Vuilleumier , P. ( 2007 ). Direct intracranial recording of body-selective responses in human extrastriate visual cortex . Neuropsychologia , 45 ( 11 ), 2621 – 2625 . 10.1016/j.neuropsychologia.2007.04.005 17499819

[b42] Powell , L. J. , Kosakowski , H. L. , & Saxe , R. ( 2018 ). Social origins of cortical face areas . Trends in Cognitive Sciences , 22 ( 9 ), 752 – 763 . 10.1016/j.tics.2018.06.009 30041864 PMC6098735

[b43] Poyo Solanas , M. , Vaessen , M. , & de Gelder , B. ( 2020 ). Computation-based feature representation of body expressions in the human brain . Cerebral Cortex , 30 ( 12 ), 6376 – 6390 . 10.1093/cercor/bhaa196 32770200

[b44] Poyo Solanas , M. , Zhan , M. , Vaessen , M. , Hortensius , R. , Engelen , T. , & de Gelder , B. ( 2018 ). Looking at the face and seeing the whole body. Neural basis of combined face and body expressions . Social Cognitive and Affective Neuroscience , 13 ( 1 ), 135 – 144 . 10.1093/scan/nsx130 29092076 PMC5793719

[b45] Raman , R. , Bognar , A. , Ghamkari Nejad , G. , Taubert , N. , Giese , M. , & Vogels , R. ( 2023 ). Bodies in motion: Unraveling the distinct roles of motion and shape in dynamic body responses in the temporal cortex . Cell Reports , 42 ( 12 ), 113438 . 10.1016/j.celrep.2023.113438 37995183 PMC10783614

[b46] Schwiedrzik , C. M. , Zarco , W. , Everling , S. , & Freiwald , W. A. ( 2015 ). Face patch resting state networks link face processing to social cognition . PLoS Biology , 13 ( 9 ), e1002245 . 10.1371/journal.pbio.1002245 26348613 PMC4562659

[b47] Stekelenburg , J. J. , & de Gelder , B. ( 2004 ). The neural correlates of perceiving human bodies: An ERP study on the body-inversion effect . Neuroreport , 15 , 777 – 780 . https://doi:.org/10.1097/01.wnr.0000119730.93564.e8 15073513 10.1097/00001756-200404090-00007

[b48] Swann , P. , Pichon , S. , de Gelder , B. , & Grèzes , J. ( 2012 ). Threat prompts defensive brain responses independently of attentional control . Cerebral Cortex , 22 ( 2 ), 274 – 285 . 10.1093/cercor/bhr060 21666127

[b49] Taubert , J. , Ritchie , J. B. , Ungerleider , L. G. , & Baker , C. I. ( 2022 ). One object, two networks? Assessing the relationship between the face and body-selective regions in the primate visual system . Brain Structure & Function , 227 ( 4 ), 1423 – 1438 . 10.1007/s00429-021-02420-7 34792643

[b50] Taylor , J. C. , Roberts , M. V. , Downing , P. E. , & Thierry , G. ( 2010 ). Functional characterisation of the extrastriate body area based on the N1 ERP component . Brain and Cognition , 73 ( 3 ), 153 – 159 . 10.1016/j.bandc.2010.04.001 20546987

[b51] Thierry , G. , Pegna , A. J. , Dodds , C. , Roberts , M. , Basan , S. , & Downing , P. ( 2006 ). An event-related potential component sensitive to images of the human body . NeuroImage , 32 ( 2 ), 871 – 879 . 10.1016/j.neuroimage.2006.03.060 16750639

[b52] Tomassini , A. , Ambrogioni , L. , Medendorp , W. P. , & Maris , E. ( 2017 ). Theta oscillations locked to intended actions rhythmically modulate perception . eLife , 6 , e25618 . 10.7554/eLife.25618 28686161 PMC5553936

[b53] Trujillo , L. T. , & Allen , J. J. B. ( 2007 ). Theta EEG dynamics of the error-related negativity . Clinical Neurophysiology , 118 ( 3 ), 645 – 668 . 10.1016/j.clinph.2006.11.009 17223380

[b54] van Heijnsbergen , C. C. , Meeren , H. K. , Grèzes , J. , & de Gelder , B. ( 2007 ). Rapid detection of fear in body expressions, an ERP study . Brain Research , 1186 , 233 – 241 . 10.1016/j.brainres.2007.09.093 17996856

[b55] Veale , R. , Hafed , Z. M. , & Yoshida , M. ( 2017 ). How is visual salience computed in the brain? Insights from behaviour, neurobiology and modelling . The Royal Society , 372 ( 1714 ), 20160113 . 10.1098/rstb.2016.0113 PMC5206280 28044023

[b56] Vogels , R. ( 2022 ). More than the face: Representations of bodies in the inferior temporal cortex . Annual Review of Vision Science , 8 , 383 – 405 . 10.1146/annurev-vision-100720-113429 35610000

[b57] Wang , X. J. ( 2010 ). Neurophysiological and computational principles of cortical rhythms in cognition . Physiological Reviews , 90 ( 3 ), 1195 – 1268 . 10.1152/physrev.00035.2008 20664082 PMC2923921

[b58] Zhu , Q. , Nelissen , K. , Van den Stock , J. , De Winter , F. L. , Pauwels , K. , de Gelder , B. , Vanduffel , W. , & Vandenbulcke , M. ( 2013 ). Dissimilar processing of emotional facial expressions in human and monkey temporal cortex . NeuroImage , 66 , 402 – 411 . 10.1016/j.neuroimage.2012.10.083 23142071 PMC3625447

