# Supplementary material for: Theta activity discriminates high-level, species-specific body processes
Source: Imaging Neurosci (Camb). 2024 Apr 26;2:imag-2-00150. doi: 10.1162/imag_a_00150 (PMC12247587; doi:10.1162/imag_a_00150)
Supplement: Supplementary Material [file imag_a_00150-supp.pdf]

9. Supplementary Materials for:

Theta activity discriminates high-level, species-specific body processes

Jane Chesley [a], Lars Riecke [a], Juanzhi Lu [a], Rufin Vogels [b,c], Beatrice de Gelder [a,d,*]

[a] Department of Cognitive Neuroscience, Faculty of Psychology and Neuroscience, Maastricht University, Maastricht 6200 MD, the Netherlands

[b] Laboratory for Neuro, and Psychophysiology, Department of Neurosciences, KU Leuven Medical School, Leuven 3000, Belgium

[c] Leuven Brain Institute, KU Leuven, Leuven 3000, Belgium

[d] Department of Computer Science, University College London, London WC1E 6BT, UK

[*] Correspondence to: Room 3.009, Oxfordlaan 55, 6229 EV Maastricht, the Netherlands. Tel. +31 433881437.

E-mail address: b.degelder@maastrichtuniversity.nl (B. de Gelder).

9.1 Supplementary Analyses

9.1.1 Event-related potential analyses

ERP analyses were performed to further investigate whether the oscillatory effect might reflect evoked or induced activity. Here, the preprocessed EEG signal was baseline-corrected by subtracting the average amplitude during the interval (– 200 ~ 0 ms) pre-stimulus, and a 50 Hz notch filter was applied. For each condition, the grand-averaged ERP was calculated over the channels (n = 3; C3, CP3, and P3) identified in the post-hoc time-frequency analyses as having the maximal difference (10th percentile) between human and monkey body stimuli. To control for the neural processing of low-level visual features, the amplitude difference (normal – scramble) was calculated for each condition. The mean amplitude difference within the relevant cluster (n = 3; C3, CP3, and P3; Fig. 6C) and time window (350 – 400 ms; Fig. 7) identified in the post-hoc time-frequency analyses was statistically analyzed with the same 2×3 repeated measures ANOVA (Species: human/monkey * Category: body/face/object) as for the time-frequency analysis; see Statistical Analyses.

9.1.2 Time-frequency analyses: Alpha- and beta- band activity

Finally, to investigate whether the effect was specific to the theta-band, we applied the analysis pipeline to alpha- (8-12 Hz) and beta-band (13-30 Hz) power. Alpha- and beta-band power during the time window of interest was extracted from the preprocessed, time-frequency transformed signal (see above). Then, to localize object-level processing channels, cluster-based permutation analysis was applied to compare all normal and all scramble conditions.

9.2 Supplementary Results

9.2.1 Event-related potential results

In line with the results based on differential theta power, the interaction effect of species*category was significant ($F(2,28) = 9.3$, $p < 0.001$, $\eta_p^2 = 0.25$). The main effect of species ($F(1,28) = 10.45$, $p = 0.003$, $\eta_p^2 = 0.27$) was significant and the main effect of category ($F(2,28) = 0.55$, $p = 0.58$, $\eta_p^2 = 0.02$) was not significant. To investigate this interaction effect, three paired samples t-tests were performed to compare the effect of species on differential theta power (normal – scramble) corresponding to body stimuli, face stimuli and object stimuli, respectively. While there was a statistically significant difference in amplitude between human faces (M = -4.2, SD = 2.97) and monkey faces (M = -0.71, SD = 2.85; $t(28) = -4.86$, $p = 0.003$), there was no significant difference in amplitude between human bodies (M = -2.11, SD = 2.9) and monkey bodies (M = -1.75, SD = 2.7; $t(28) = -0.59$, $p = 0.279$), nor between human objects (M = -2.63, SD = 2.72) and monkey objects (M = -1.45, SD = 2.75; $t(28) = -1.7$, $p = 0.075$), mismatching the results based on differential theta power (Fig. S1). This indicates that the species effect on body processing was reflected in theta oscillations rather than stimulus phase-locked activity. The face-specific species effect on the ERP may suggest there are stimulus-locked differences in the higher-order visual representations of human versus monkey faces. Future research is needed to better understand the dynamics of species-specific face processing.

9.2.2 Time-frequency results: Alpha- and beta- band activity

There was no significant difference between normal and scramble conditions at any clusters of electrodes during the time window of interest in the alpha- or beta-bands (Fig. S2).

9.3 Supplementary Figures and Tables

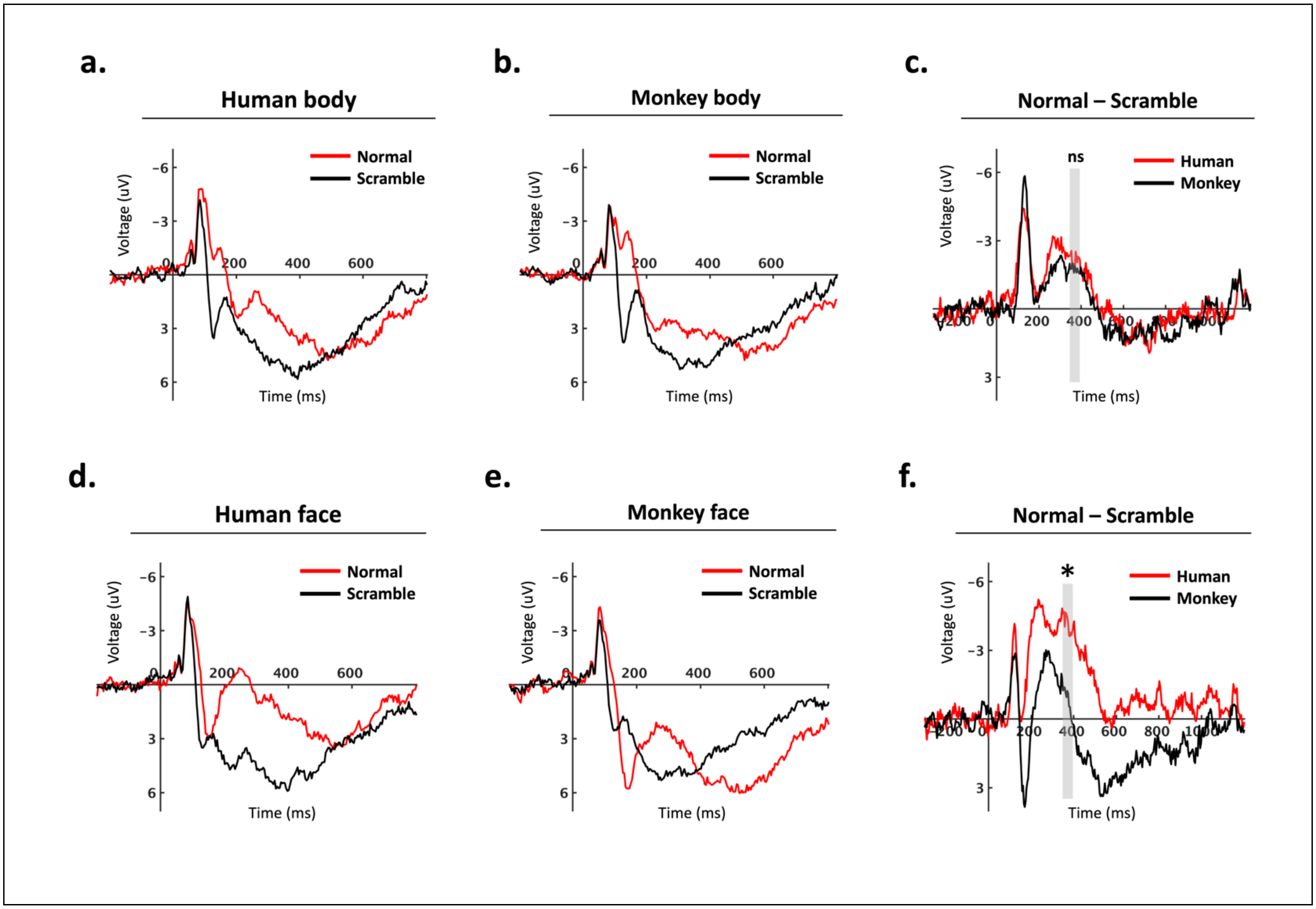

Figure S1. Grand-averaged ERP waveforms per condition, calculated by averaging the data at electrodes C3, CP3, and P3. (C) Difference waveforms (normal – scramble) shown separately for human and monkey body stimuli. (F) Difference waveforms (normal – scramble) shown separately for human and monkey face stimuli. The grey box highlights the time window (350 – 400 ms) used for statistical analyses. There was no significant difference between scramble-controlled human and monkey body stimuli (C). There was a significant difference between scramble-controlled human and monkey face stimuli, as indicated with an asterisk (F).

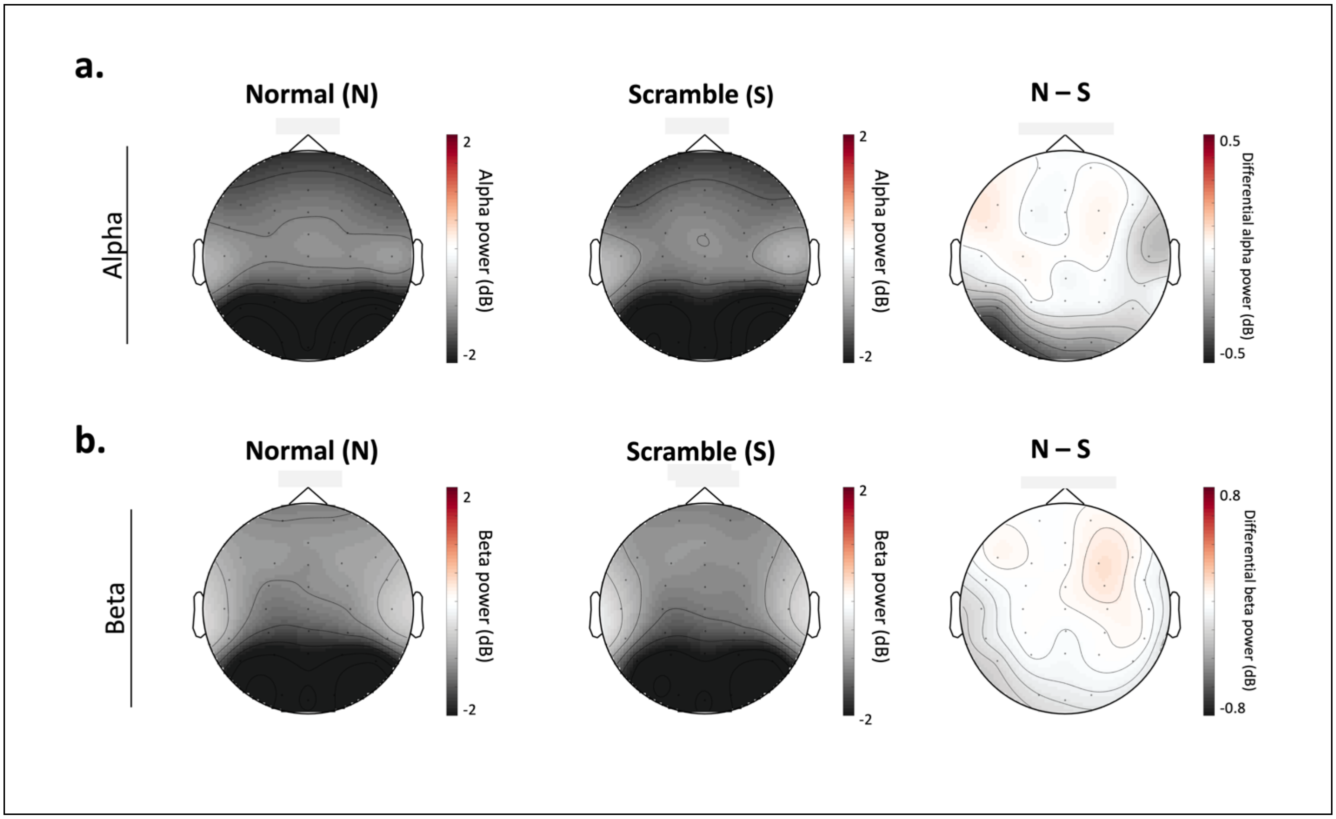

Figure S2. (A) Alpha power (8 – 12 Hz) and (B) beta power (13 – 30 Hz) during the time window of interest (200 – 550 ms post-stimulus) for all normal (left) and all scramble (middle) conditions. The difference in power (normal – scramble) is represented on the right. Cluster-based permutation analysis revealed no significant difference between all normal and all scramble conditions within any clusters of electrodes in alpha- or beta-band frequencies.
